# Mental Health System Responsiveness during COVID-19 in People with Pre-Existing Psychiatric Disorders: Experiences from Iran

**DOI:** 10.3390/epidemiologia4010008

**Published:** 2023-02-20

**Authors:** Maryam Zabihi Poursaadati, Samaneh Hosseinzadeh, Masoomeh Maarefvand, Jafar Bolhari, Jagdish Khubchandani

**Affiliations:** 1 Department of Social Work, School of Social Health, University of Social Welfare and Rehabilitation Sciences, Tehran 1985713871, Iran; 2 School of Rehabilitation Sciences, University of Social Welfare and Rehabilitation Sciences, Tehran 1985713871, Iran; 3 Department of Psychiatry, Spiritual Health Research Center, Iran University of Medical Sciences, Tehran 1449614535, Iran; 4 Department of Public Health, College of Health Education and Social Transformation, New Mexico State University, Las Cruces, NM 88011, USA

**Keywords:** mental health system, responsiveness, COVID-19, psychiatric disorder

## Abstract

Mental health system responsiveness (MHSR) is one of the important indicators in measuring the performance of mental health systems. Recognizing this function can be effective in responding appropriately to the needs of People with Pre-Existing Psychiatric Disorders (PPEPD). This study aimed to investigate MHSR during the COVID-19 period in PPEPD in Iran. Using stratified random sampling, 142 PPEPD who were admitted to a Psychiatric Hospital in Iran one year before the onset of the COVID-19 pandemic were recruited for this cross-sectional study. Participants completed a demographic and clinical characteristics questionnaire as well as a Mental Health System Responsiveness Questionnaire through telephone interviews. The results show that the indicators of prompt attention, autonomy, and access to care were reported as the worst-performing and the confidentiality indicator as the best-performing. The type of insurance affected the access to care and the quality of basic amenities. MHSR has been reported to be poor in Iran in general and this problem worsened during the COVID-19 pandemic. Considering the prevalence of psychiatric disorders in Iran and the degree of disability of these disorders, structural and functional changes are needed for adequate MHSR.

## 1. Introduction

COVID-19 has become the worst global health crisis of the century with more than 800 million cases and 7 million deaths worldwide by February 2023 [1] and has led to significant disruptions in the social, economic, and political climate and, most significantly, human health [2]. Governments around the world have been forced to adopt serious measures to curb COVID-19 infections, including, but not limited to, social distancing, quarantine, lockdown, mass-media campaigns, non-essential workplace and school closures, as well as transportation shutdowns [3,4]. These measures have caused many negative psycho-socioeconomic consequences for individuals, communities, and societies.

A plethora of evidence shows that lockdowns and quarantines were associated with traumatic stress symptoms, depression, anxiety, sleep problems, and loneliness [5]. Having family members together for long hours increased tensions and increased the likelihood of domestic violence [6]. Job closures reduced job security, and many people lost their jobs or faced declining incomes during this time [7]; these factors consistently correlate with depressive symptomatology.

Pandemics and epidemics have often posed a disproportionately higher and more negative impact on vulnerable and marginalized groups [8,9]. However, healthcare workers were also exposed to psychological vulnerabilities resulting from the consequences of COVID-19, which affected their service delivery [10,11]. People with Pre-Existing Psychiatric Disorders (PPEPD) may be among the most vulnerable populations [12]. Evidence suggests that these people are more susceptible to the effects of major life stressors and events, including pandemics [13], which can lead to relapse or worsening of symptoms [14]. During the COVID-19 pandemic, extensive closures and transportation restrictions reduced PPEPD access to medical visits, consultations, and medications, as well as disrupted existing sources of social support [12,14,15,16]. Small social networks also limited opportunities to obtain support from friends and family members [12]. These people were at higher risk of infections (including COVID-19) due to cognitive impairment, frailty, unhealthy lifestyles and health risk behaviors, little awareness of infection risk, and lower efforts regarding personal protection [14,17]. Additionally, residential instability and homelessness could also raise the risk of infection and make it harder to identify, follow up, and treat those who are infected [18].

Measuring the performance of health systems can identify their strengths and weaknesses, especially in times of crisis. One of the most important indicators for measuring the performance of the health system is responsiveness. Responsiveness is one of the main goals of any reasonable health system and is defined as how the health system fulfills the expectations of the population as it relates to the non-health-enhancing aspects of the health system. It includes eight elements: dignity, confidentiality, autonomy, clear communication, prompt attention, social support, basic amenities, and choice of provider [19]. The concept of responsiveness has foundations in the principles of human rights, both of which pursue the goal of equitable distribution of health services with respect for human rights and dignity [20]. Although this index measures non-medical aspects of the health system, it pays attention to dimensions that have a serious impact on health and medical treatment [21]. This is in contrast to patient satisfaction, which has been proven to be unreliable in measuring health system performance, while the concept of responsiveness provides results that are more reliable, credible, and sensitive to change [22]. This tool is designed to respond to the health system in general, but studies show that it also reflects the specific expectations of people with mental health needs who interact with providers and systems [22,23,24]. Responsiveness includes main components such as prompt attention, which means establishing proper communication between service providers and patients. This communication should be such that patients have enough time to express their problems and can ask questions about the disease and the treatment process. Further, in this communication process, the providers follow up on the patient’s condition accurately and continuously. The other component of responsiveness is respect, which means that all the members of the service provider team should treat patients respectfully and without prejudices and stigmas and take special care of their needs and characteristics, respecting the privacy of patients. Autonomy expresses the equality of the relationship between the patient and the therapist. This means that patients can freely choose their therapist and service providers and actively participate in the decision-making and treatment process. Clear communication means providing information about the disease and treatment to patients in a comprehensible manner [23].

Few studies have assessed responsiveness in the Middle East. For example, studies in Iran have examined the responsiveness of the health system in public hospitals. The indicators of social support [25,26] and confidentiality [25,26,27] are the best, and indicators of autonomy [25,27] in the inpatient wards and prompt attention [27,28], quality of basic facilities [28], and autonomy [25,27] in the outpatient wards were the lowest. Forouzan et al. (2016) studied the dimensions of responsiveness in people with psychiatric disorders who were referred to the outpatient clinics and indicated that the indicators of confidentiality and dignity had the best performance and the indicators of autonomy, access to care, and quality of basic amenities had the worst performance. On average, 47% of the study participants reported experiencing poor responsiveness [23]. During the COVID-19 pandemic, little is known about the responsiveness of the healthcare systems for this large and vulnerable population of PPEPD, especially in Iran, despite the rapid changes due to the pandemic, declining rates of services and hospitalizations in psychiatric hospitals, and repurposing of psychiatric hospitals for use by COVID-19 patients or isolation of patients due to COVID-19 infections. Furthermore, the health system of the country was mobilized to respond to the epidemic crisis in Iran, and the needs of the PPEPD were neglected. In times of crisis, marginalized groups are more likely to be harmed than at any other time. Estimating the level of risk can help health system decision makers and planners to design and revise their protocols and guidelines according to the situation of the target group. Considering that the level of responsiveness of the health system to PPEPD in critical periods in Iran has not been investigated, thus, the purpose of this study was to measure the Mental Health System Responsiveness (MHSR) during the pandemic from the perspective of PPEPD.

## 2. Materials and Methods

### 2.1. Setting

This study was conducted at Razi Psychiatric Hospital (RPH), which is a public university hospital in Tehran, Iran. This hospital has 1375 beds and is the largest psychiatric hospital in Iran and the Middle East. In this hospital, people with various psychiatric disorders in all age groups receive outpatient and inpatient medical and rehabilitation services from multidisciplinary teams including psychiatrists, nurses, social workers, psychologists, occupational therapists, physiotherapists, and other specialists.

During the pandemic, when Iran was facing high infection and mortality rates, RPH was forced to reduce the number of admissions to minimize the possibility of transmission in the wards. Patients were tested for COVID-19 before admission and transferred to the quarantine ward if positive. Inpatient wards that used to receive between 40 and 45 patients before COVID-19, were reduced to 25 beds. Further, to comply with protocols, patient visits were canceled.

### 2.2. Study Population

This study was done retrospectively. Participants in this study were selected from a pool of individuals who were hospitalized in the RPH one year before the onset of the pandemic in Iran, i.e., from February 2019 to February 2020. For this purpose, the list of all hospitalized patients in RPH was extracted from the hospital information system for the study period. Patients who were referred from social support organizations or the judicial system were removed from the list due to a lack of contact information. In total, 4125 PPEPD were included as potential participants in our study. Then, the number of PPEPD per month was calculated based on the total acceptance ratio in that month and the participants were selected by stratified random sampling method. The completion of the questionnaires was done from July to September 2021 through telephone calls with the participants. One of the members of the research team called the available number and provided the necessary explanations about the research to the patient and/or primary caregiver. If the patient and/or primary caregiver were willing to participate in the study, a time for an interview was set. Due to COVID-19 limitations, the questionnaire was completed by telephone. Three social workers were trained to deploy the questionnaire. To measure the MHSR during the pandemic from the perspective of PPEPD, we included individuals who needed psychiatric services during the twelve months before the start of the study (so, all patients who did not need to receive psychiatric services during the same period were excluded from our study). Using sampling with replacement, we selected eligible patients at random from the list and replaced another patient with the ones who did not meet the inclusion criteria.

According to the previous study [29], the standard deviation of the overall responsiveness score is 0.6. Considering a power of 80% (z = 0.85), a confidence level of 95% (z = 1.96), and a difference of 0.2, the average score of the response variable before and after COVID-19 is at least d = 0.2, the sample size is 142 people. We stopped sampling when 142 questionnaires were completed.

### 2.3. Instrument and Data Analysis

In this study, the Mental Health System Responsiveness Questionnaire (MHSRQ), which is derived from the WHO tool and was validated in 2016 on the Iranian population with psychiatric disorders, was used [26]. This standardized questionnaire had 42 health system responsiveness closed-ended Likert scale questions that were grouped under nine domains that have ordinal response categories, namely: access to care (five questions), clear communication (three questions), confidentiality (three questions), dignity (four questions), prompt attention (eight questions), autonomy (six questions), effective care (five questions), quality of basic amenities (four questions), and social support (four questions). All questions had similarly ordered 4-point response options (always, usually, sometimes, never) or 5-point (mainly: very good, good, moderate, bad, very bad) response options. Based on WHO’s study, verbal response options for each question were coded to numeric values, 1 corresponding to the worst, and 4 or 5 to the best response options [30]. A further summary score for “overall responsiveness” was obtained by calculating the average scores across all eight domains. The responsiveness outcomes were then dichotomized into good responsiveness (combining the very good and good responses) and poor responsiveness (combining the moderate, bad, and very bad responses) [23]. Cronbach alpha was computed to assess the internal consistency reliability for the MHSRQ and was found to be high (α = 0.90).

Sociodemographic variables included age, gender, job status, educational status, and living status. In this study, age was measured in years, sex was categorized into male or female, and job status was categorized into employed full-time, unemployed, retired, and part-time worker. Further, educational status was categorized into primary level (<5 years of education), intermediate level (5–12 years of education), and higher education level (>12 years of education). Living status was categorized into living with a spouse or family, friends or colleagues, single/living alone, and homeless. Some clinical characteristics were also asked, including the type of disorder, duration of illness, frequency of hospitalization, substance use status, COVID-19 infection history, and adherence to health protocols.

Due to the lack of normal distribution of data, we applied the Spearman correlation test, Mann-Whitney U, and Kruskal–Wallis H tests to check the bivariate association between subscale and overall responsiveness and sociodemographic characteristics. All analyses were performed using SPSS version 24 and *p*-values < 0.05 were considered significant.

### 2.4. Ethics

The study was approved by the Ethics Committee of the University of Social Welfare and Rehabilitation Sciences, Tehran, Iran (case number: 1397.131). Due to COVID-19 restrictions, informed consent was obtained orally and by telephone from participants. To respect the rights of the participants, the questionnaires were completed and analyzed anonymously, participation in the research was voluntary, and participants could withdraw from the research at any time.

## 3. Results

A total of 255 numbers were called from the list; 113 people did not complete the questionnaire for the following reasons: 31 people did not need mental health care in the past year; 27 people were reluctant to participate in the study; 18 people stated that their patient was in a residential care center; 14 patients had died; 7 patients were in prison; 4 people were homeless and family members were unaware of them; and 12 numbers were also wrong. Among the total of 142 study participants, 55 participants (38.7%) had experienced hospitalization in the past year, while 40 participants (28.2%) who experienced a relapse needed only outpatient treatment. A total of 21 participants (14.8%) who had experienced relapse and had been hospitalized by a psychiatrist did receive inpatient treatment, and 26 participants (18.3%) did not seek treatment despite the relapse at all. The mean and standard deviation of disease duration among participants was 13.89 ± 8.27 (min: 5 y; max: 40 y). Most of the participants were male (69.7%), between 30 and 39 years old (35.2%), and did not have a job (59.1%). Bipolar disorder was the most reported disorder among participants (30.3%). The majority of participants had less than 10 years of disease history (45.8%) and had experienced between one and five times of psychiatric hospitalization (62%). Details of the sociodemographic characteristics of the study participants are presented in Table 1.

Table 2 shows that, except for the confidentiality subscale, the rest were reported as poor. The subscales of autonomy, prompt attention, and access were the worst performing, and confidentiality was the best. Further, patients who had been hospitalized in the past year reported the quality of basic amenities close to good performance, which is the next stage after confidentiality.

To examine the relationship between clinical and demographic variables with subscales and the overall score of mental health system responsiveness, the Mann-Whitney test was used. The results showed the mean values of clear communication (Z = −3.169; *p*-value = 0.002), confidentiality (Z = −2.459; *p*-value = 0.014), prompt attention (Z = −2.324; *p*-value = 0.020), effective care (Z = −2.665; *p*-value = 0.008), and quality of basic amenities (Z = −2.542; *p*-value = 0.011) subscales and total Score (Z = −2.585; *p*-value = 0.010) were significantly different between male and female patients, and females reported the worse experience in these subscales. The mean subscale of social support in hospitalized patients (Z = −2.028; *p*-value = 0.043) was significantly lower than that in outpatients.

The quality of the basic amenities subscale was significantly lower in patients covered by support organizations than in those who are not covered (Z = −2.328; *p*-value = 0.020). The mean subscales of prompt attention (Z = −2.029; *p*-value = 0.042) and social support (Z = −3.133; *p*-value = 0.002) in patients who were infected by COVID-19 were significantly lower than in patients who did not report the infection.

Further, participants who adhered to at least one of the COVID-19 health protocols (wearing a mask, washing hands, and social distancing) reported better scores on access to care (Z = −2.394; *p*-value = 0.017), confidentiality (Z = −2.844; *p*-value = 0.004), prompt attention (Z = −4.065; *p* value = 0.000), effective care (Z = −4.027; *p* value = 0.000), quality of basic amenities (Z = −3.677; *p*-value = 0.000), and social support (Z = −3.658; *p*-value = 0.000) subscales and the total score (Z = −4.428; *p*-value = 0.000) than patients who did not.

Participants who had substance use reported lower scores on the clear communication (Z = −3.36; *p*-value = 0.001) and dignity (Z = −2.18; *p*-value = 0.029) subscales than those who did not.

Further, Kruskal–Wallis H test result showed that there was a significant relationship between employment and effective care (χ^2^ = 18.127; *p*-value = 0.001) subscale as well as overall score (χ^2^ = 11.680; *p*-value = 0.020). Type of insurance has a significant relationship with the access to care (χ^2^ = 10.51; *p*-value = 0.015) and quality of basic amenities (χ^2^ = 8.56; *p*-value = 0.036) subscales. The level of education has a significant relationship with the subscales of clear communication (χ^2^ = 7.51; *p*-value = 0.023) and confidentiality (χ^2^ = 6.17; *p*-value = 0.046); furthermore, the source of income has a significant relationship with the subscales of clear communication (χ^2^ = 12.25; *p*-value = 0.002) and confidentiality (χ^2^ = 6.18; *p*-value = 0.045).

The results of the Spearman correlation test showed that there was no significant relationship between the variables of age, number of hospitalization, and duration of illness with subscales and overall scores of the questionnaire.

## 4. Discussion

This cross-sectional study aimed to investigate the MHSR during the COVID-19 pandemic from the perspective of PPEPD in Iran. The results of this study showed that MHSR in Iran during the COVID-19 pandemic in the subscales of autonomy, prompt attention, and clear communication were in the worst state and the subscale of confidentially was in a good state. Women evaluated these subscales more poorly than men. In the subscale of social support, inpatients compared to outpatients and patients with COVID-19 compared to non-COVID-19 evaluated the situation as poorer. Further, the results showed that the patients with COVID-19 reported the prompt attention scale poorer than the non-infected patients. Further, people who adhered to the health rules related to the COVID-19 pandemic reported better access to care. Participants with substance use also scored poorly on the clear communication subscale.

The results of this study, following Forouzan’s earlier research in Iran [23], showed the indicators of autonomy and access to care had the worst status among the subscales and the confidentiality indicator had the best performance. In the present study, unlike Forouzan’s earlier research in Iran before the pandemic, the quality of basic facilities for patients was in near-good condition. Further, although the dignity indicator had an acceptable score in the previous study, it has been reported as weak in this study.

The mental health system in Iran is limited to psychiatric hospitals and psychiatric beds in general hospitals. In the last decade, projects such as the integration of the mental health system in primary health care, the establishment of comprehensive mental health centers in metropolitan areas, and the provision of home visit services and follow-up after discharge have been implemented in a limited and regional manner, but they did not expand due to a lack of structural and budgetary support [31,32]. People with psychiatric disorders are now forgotten after discharge from the hospital until the next relapse occurs. In pandemic conditions, this forgetfulness intensified because the capacity of the beds was reduced. These conditions significantly reduced patient and family access to the mental health system.

The results of previous research conducted in general hospitals [25,26,27,28] also showed that autonomy and prompt attention have the worst performance among the indicators, which was also confirmed in this study. It seems that despite the expansion of attention to patient rights in the treatment process in recent years in the Iranian health system, patient participation in the decision-making process for treatment is still a major challenge. While this was certainly due to the pandemic, there are greater challenges for people with mental health issues as these populations are incapacitated due to some symptoms such as decreased insight and cognitive impairment, leading to a lower tendency to seek care, and their right to participate is also ignored.

Although in the previous study from before the pandemic in Iran [23] wherein access to care had one of the worst-performing scores, in the present study, the percentage of people who reported this indicator as poor increased remarkably (from 31.9% to 78.2%). Quarantine appears to have reduced access to health care for PPEPD. In such situations, some countries quickly devised strategies such as the expansion of services through unique health interventions and remote technologies [33,34]. However, in Iran, due to poor coverage and speed of the internet, low skills of people working with social networks, and lack of access to smartphones or the internet, the remote interventions did not spread enough to meet the needs of PPEPD.

Gross inequities occur within the population of PPEDP. For example, in this study, women with psychiatric disorders reported poorer MHSR than men. Given the lack of resources, lower-income, and education and rights for women, they are generally exposed to medical and social discrimination; during the pandemic, women shared a greater burden of inequity and discrimination. Women have a smaller share of psychiatric beds in Iran and on the other hand, the stigma of the disease is more pronounced for them. Future studies should explore gender-based differences in MHSR and gender-sensitive interventions for PPEPD in Iran.

Previous studies have examined the health system responsiveness in primary health care [26] and public and private general hospitals [25] and have reported social support indicators as good, but the present study showed that patients who were admitted described poor social support. It seems that the restriction imposed by medical centers for patients and their families, which was done to prevent the spread of COVID-19 infections, could have played a role in lower social support scores. In this study, patients who did not adhere to health protocols reported all subscales in a worse condition, and non-compliance with protocols had reduced the mental health system’s responsiveness to PPEPD; although the general policy of the health system seemed to be to adhere to protocols to reduce the rate of transmission and infection, PPEPD due to some symptoms such as cognitive impairment at the time of relapse were not aware of the danger and the need for self-care.

People with psychiatric disorders are generally exposed to social exclusion and discrimination due to the stigma of psychiatric disorders. COVID-19 infection can also add to this stigma and lead to a smaller support network for these people, which can have a negative long-term impact on the social status and physical and mental health of patients. Given the pandemic-related limited access to treatment, it is obvious that the symptoms of psychiatric disorders will recur and relapse for many, followed by a decrease in the individual’s skills in self-care, and as a result, the patient will not be able to follow the health protocols to prevent COVID-19 and this will lead to a vicious cycle of negative outcomes that have interrelated causal factors.

Another factor that seems to be effective in MHSR is insurance coverage. Although in the last decade, the Iranian Ministry of Health and Medical Education has tried to provide primary insurance coverage to the public through Iranian Health Insurance, insurance coverage for mental health services does not meet the needs of patients [32,35]. Various insurances cover between 90 and 97% of the costs of hospitalization in a public psychiatric hospital, but only cover 56 days of hospitalization per year and do not cover other psychiatric services such as monthly visits, medications, and rehabilitation services [36]. However, due to the lack of after-discharge services and defective treatment cycles, people with severe psychiatric disorders experience several relapses and need continuous services.

In this study, one-third of the participants reported substance use. Substance use leads to a more complex disease profile and is one of the important risk factors for relapse [37]. As mentioned above, relapse can lead to reduced insight, cognitive deficits, and reduced self-care in the patient, and high-risk behaviors cause the patient to be exposed to COVID-19. Factors such as unemployment or unstable jobs, along with poor insurance coverage, make it difficult for PPEPD to pay for physical and mental health care and leads to treatment gaps. On the other hand, social support for PPEPD in Iran does not meet their needs. As seen in this study, the majority of PPEPDs are not covered by any of the support organizations. In general, in Iran, the State Welfare Organization oversees supporting people with severe psychiatric disorders. Pension is the most important service that this organization provides for this group. The amount of the pension is barely enough to pay for a patient’s medication. Inpatient and outpatient rehabilitation centers operate services for people with severe psychiatric disorders under the supervision of this organization. However, all these centers are private, and the patient and the family have to pay to benefit from the services of these centers. Sometimes a 24-h rehabilitation center costs 10 to 20 times the monthly pension. On the other hand, the number of daily rehabilitation centers is insufficient, and during the COVID-19 pandemic, many of these centers reduced or stopped their activities. Lack of social support leads to more marginalization of PPEPD. Studies have also shown that the informal support network for PPEPD is limited [38,39,40], and this issue was exacerbated during this pandemic, due to the policy of social distancing. As a result, PPEPD faced severe restrictions on support from the formal and informal support networks, which led them to experience further isolation.

This study has several potential limitations. First, the data collection in this study coincided with the most severe pandemic peak in Iran, and as a result, it was not possible to meet the participants. Initially, the questionnaire was designed online, but a significant number of participants did not have access to a smartphone or could not complete the questionnaire due to low literacy. Therefore, the questionnaires were completed by telephone. Since desirability bias may occur in this way, interviewers were trained to minimize this bias. Some of the questions in this questionnaire required a reminder of the past, which may have led to a recall bias. To reduce this bias, some information was checked with the hospital information system. Given the cross-sectional study design, cause-and-effect relationships cannot be established among study variables. Finally, the results of this study suffer from all traditional limitations of reliability and validity associated with survey study designs. Despite these limitations, the study has many strengths. First, this is one of the few unique assessments of MHSR during the pandemic. Second, our sample size was reasonable with a diverse group of individuals representative of a large metropolitan population in Iran. Finally, the use of a previously validated tool to assess MHSR adds credibility to our findings.

## 5. Conclusions

The performance of MHSR in Iran before the pandemic was reported to be poor in most indicators, but in this study, it was found that this performance had a major and adverse decline during the COVID-19 pandemic. In this study, indicators such as prompt attention, autonomy, and access to care were reported as the worst-performing. Adherence to the principles of professional ethics, observance of the Patients’ Rights Charter, and the development of an approach to respect human dignity can play a more prominent role in medical education and treatment and improve the two indicators of prompt attention and autonomy. Major structural and functional changes are warranted in Iran to cater to the needs of individuals with mental health and behavioral health needs. For example, concerning the access to care index, there is a need for a change in the pricing and payment of the mental health system and in the development of infrastructure. Increasing the share of the mental health system in the public health expenditure and the GDP is warranted. Especially, for rehabilitation and after-discharge services, the development of remote interventions, the development of community-based interventions through capacity building, community empowerment in mental health care, and post-discharge services are strategies for improving the MHSR in Iran.

## Figures and Tables

**Table 1:** Sociodemographic characteristics of the study group.

Variable		Participant (%)
Age (Mean ± SD: 36.85 ± 10.39)	20–29	43(30.3%)
	30–39	50 (35.2%)
	40–49	29 (20.4%)
	50<	20 (14.1%)
Duration of illness (years) (Mean ± SD: 13.89 ± 8.28)	1–10	65 (45.8%)
	11–20	53 (35.2%)
	20<	24 (16.9%)
Number of hospitalizations (Mean ± SD: 5.75 ± 4.74)	1–5	88 (62%)
	6–10	32 (22.5%)
	10<	22 (15.5%)
Gender	Female	43 (30.3%)
	Male	99 (69.7%)
Job	Employed full time	6 (4.2%)
	Retired	9 (6.3%)
	Unemployed	84 (59.1%)
	Part-time worker	43 (30.3%)
Education	Primary level	12 (8.5%)
	Intermediate level	91 (64.1%)
	Higher education level	39 (27.5%)
Living Status (with whom)	Spouse or family	111 (78.2%)
	Friends or colleagues	9 (6.3%)
	Single/alone	21 (14.8%)
	Homeless	1 (0.7%)
Type of disorder	Schizophrenia	26 (18.3%)
	Depression	28 (19.7%)
	Bipolar Disorder	43 (30.3%)
	Obsessive-compulsive disorder	6 (4.2%)
	Substance use disorder	14 (9.9%)
	Comorbidity of psychiatric disorder and substance use	25 (17.6%)
Source of income	Employment	78 (54.9%)
	Retirement	35 (24.6%)
	Unofficial sources	29 (20.4%)
Formal social support	Yes	63 (44.4%)
	No	79 (55.6%)
COVID-19 infection history	Yes	117 (82.4%)
	No	25 (17.6%)
Commitment to health protocol	Yes	85 (59.9%)
	No	57 (40.1%)
Substance use	Yes	43 (30.3%)
	No	95 (66.9%)
	Unknown	4 (2.8%)

**Table 2:** Descriptive statistics of subscales of the Mental Health System Response Questionnaire.

Subscales of the MHSRQ	Min Score	Max Score	Mean Total	Mean Male	Mean Female	Poor N (%)	Good N (%)
Access to care	9	17	12.41	12.63	12.31	111 (78.2%)	30 (21.1%)
Clear communication	2	13	7.66	8.31	7.38	105 (73.9%)	35 (24.6%)
Confidentiality	4	13	10.26	10.67	10.08	26 (18.3%)	114 (80.3%)
Dignity	5	17	10.64	11.19	10.41	88 (62.1%)	51 (35.9)
Prompt attention	3	33	17.65	19.21	16.99	119 (83.8%)	21 (14.8%)
Autonomy	2	24	12.92	13.12	12.84	125 (88%)	15 (10.6%)
Effective care	5	18	12.39	13.12	12.07	99 (69.7%)	39 (27.5%)
Quality of basic amenities	8	20	13.58	14.52	13.18	77 (54.2%)	62 (43.7%)
Social support	3	19	7.38	8.34	6.99	89 (62.7%)	9 (6.3%)

## Data Availability

Research data can be provided upon qualified and allowable requests from investigators.
